# Resveratrol Attenuate Myocardial Injury by Inhibiting Ferroptosis *Via* Inducing KAT5/GPX4 in Myocardial Infarction

**DOI:** 10.3389/fphar.2022.906073

**Published:** 2022-05-24

**Authors:** Jing Liu, Mingming Zhang, Chaoshi Qin, Zikuan Wang, Jianghong Chen, Rui Wang, Jianqiang Hu, Qing Zou, Xiaolin Niu

**Affiliations:** Department of Cardiology, Tangdu Hospital, Air Force Medical University, Xi’an, China

**Keywords:** myocardial infarction, myocardial injury, ferroptosis, resveratrol, KAT5, GPX4

## Abstract

Myocardial infarction (MI) is a coronary artery-related disease and ranks as the leading cause of sudden death globally. Resveratrol (Res) is a bioactive component and has presented antioxidant, anti-inflammatory and anti-microbial properties. However, the effect of Res on ferroptosis during MI progression remains elusive. Here, we aimed to explore the function of Res in the regulation of ferroptosis and myocardial injury in MI. We observed that the treatment of Res attenuated the MI-related myocardium injury and fibrosis in the rats. The expression of collagen 1 and α-SMA was induced in MI rats, in which the treatment of Res could decrease the expression. Treatment of Res suppressed the levels of IL-6 and IL-1β in MI rats. The GSH levels were inhibited and MDA, lipid ROS, and Fe^2+^ levels were induced in MI rats, in which the treatment of Res could reverse the phenotypes. Meanwhile, the expression of GPX4 and SLC7A11 was reduced in MI rats, while the treatment of Res could rescue the expression in the model. Meanwhile, Res relieved oxygen-glucose deprivation (OGD)-induced cardiomyocyte injury. Importantly, Res repressed OGD-induced cardiomyocyte ferroptosis *in vitro*. Mechanically, we identified that Res was able to enhance GPX4 expression by inducing KAT5 expression. We confirmed that KAT5 alleviated OGD-induced cardiomyocyte injury and ferroptosis. The depletion of KAT5 or GPX4 could reverse the effect of Res on OGD-induced cardiomyocyte injury. Thus, we concluded that Res attenuated myocardial injury by inhibiting ferroptosis *via* inducing KAT5/GPX4 in myocardial infarction. Our finding provides new evidence of the potential therapeutic effect of Res on MI by targeting ferroptosis.

## Introduction

Myocardial infarction (Fang et al., 2020) is a coronary artery-related disease that severely threatens human life, and ranks as the leading cause of sudden death globally (Anderson and Morrow, 2017). The common pathological factors of MI include abnormal supplement of myocardial oxygen or coronary atherothrombosis (Gulati et al., 2020). When the atherosclerotic thrombosis is raptured, the released plaque causes accumulation of platelets, which leads to occlusion of coronary artery, and subsequent myocardial ischemia and necrosis (Boateng and Sanborn, 2013). On the other hand, the lack of nutrients and oxygen leads to inflammatory response, and death of cardiomyocytes (Cabello et al., 2016; Reed et al., 2017). It has been well-identified that the inflammatory process is involved in the pathogenesis of MI and the pro-inflammatory cytokines, including interleukin‐1β (IL‐1β) and interleukin‐6 (IL-6), were enhanced (Bezerra et al., 2017; Liu et al., 2019). Meanwhile, MI is associated with left ventricular (Liu et al., 2021) remodeling and is featured by the cardiac fibrosis, leading to the increases of fibrosis markers, such as collagen I and α-smooth muscle actin (α-SMA) (Wang et al., 2020). Although the exploration of pathogenesis of MI has made great progress over the past years, the incidence of MI still steadily increases (Chen et al., 2017). Therefore, it is urgent to develop therapeutic manners for MI.

Integrated Traditional Chinese and Western medicine plays an important role in the treatment of various diseases (Bonnefont-Rousselot, 2016). Noteworthy, the Traditional Chinese medicine has been widely applied in the prevention and treatment of metabolic syndrome (Dong-mei et al., 2013; Lu et al., 2019). Res is a bioactive component first discovered in *Veratrum gandiflorum* roots, and has been widely studies in diseases treatment, owing to its antioxidant, anti-inflammatory and anti-microbial properties (Chaplin and Mercader, 2018; Huang et al., 2019). Moreover, Res has been discovered to modulate multiple molecular targets, such as AMP-activated protein kinases, nuclear factor erythroid 2-associated factor 2, nuclear factor—(NF-) κB and endothelial nitric oxide synthase (Davies and Roulleau, 2012; Price et al., 2012; Kim et al., 2018; Huang et al., 2019). Meanwhile, the oxidative stress and inflammation are important pathophysiological mechanisms of various cardiovascular diseases.

Ferroptosis is a newly discovered form of non-apoptotic cell death related to metabolic dysfunction, which leads to abnormal decomposition of glutamine during metabolism process, iron-dependent accumulation of reactive oxygen species (ROS), mitochondrial superoxide, as well as decreased mitochondrial membrane potential (Dixon et al., 2012; Koppula et al., 2020). During ferroptosis, the phospholipid glutathione peroxidase 4 (GPX4) catalyzes the reduction of peroxide such as hydrogen peroxide and organic hydroperoxides, and plays critical inhibitory role (Yang et al., 2014). In addition to iron overload, depletion of glutathione (GSH) is another crucial mechanism during ferroptosis, and the accumulation of cellular GSH is regulated by SLC7A11, which transports GSH precursor cystine in the cytosol (Fang et al., 2020). Studies have revealed that ferroptosis contributes to the death of cardiomyocytes and development of MI (Song et al., 2021; Wang and Kang, 2021). Nevertheless, the molecular mechanism of ferroptosis in the pathogenesis of MI, as well as the function of Res on ferroptosis during MI is unclear.

In this work, we aimed to explore the function of Res on MI, determined that the administration of Res alleviated MI and inflammatory response both *in vitro* and *in vivo*, and inhibited ferroptosis through recruiting KAT5 to enhance GPX4 expression. Our work presented novel mechanism of Res as a therapeutic agent for MI.

## Materials and Methods

### Cell Culture and Treatment

Rat cardiomyocytes H9c2, was obtained from the American Type Culture Collection (ATCC, United States), cultured in DMEM (Thermo, United States) supplemented with 10% fetal bovin serum (FBS, Gibco) and 1% penicillin/streptomycin, in a 37°C humidified incubator with 5% CO_2_. The small interfering RNAs targeting KAT5 (siKAT5) or GPX4 (siGPX4) were synthesized by RiboBio (China), and transfected to H9c2 cells by using lipofectamine 2000 reagent (Invitrogen, United States) in line with manufacturer’s protocol. Res was purchased from Sigma (United States) and H9c2 cells were treated with Res (50 μM) according to the references (Xu et al., 2019a; Hu et al., 2021), and the same volume of PBS was used in the Mock group. Ferrostatin-1 (Fer-1) was purchased from MCE (United States) and H9c2 cells were treated with Fer-1 (2.5 μM) according to the reference (Ning et al., 2021).

### Establishment of MI Rat Model

All animal experiments were authorized by the Ethics Committee of the Tangdu Hospital. The SD rats aged 8-weeks old were brought from Beijing Vital River laboratory (China). To establish MI model, the rats were administrated with left anterior descending (LAD) ligation by using 6–0 silk suture in accordance with standard protocol (Huang et al., 2020). The rats in treatment group received injection of Res (20 mg/days) surrounding the infarcted heart area. Rats in the control group received operation without ligation, and were injected with same volume of PBS instead of resveratrol. Transthoracic two-dimensional (2D) echocardiography was performed to evaluate the heart function 7 days after the operation by using a Vevo 2,100 ultrasound system. Left ventricular ejection fraction (LVEF), left ventricular fractional shortening (LVFS), left ventricle anterior wall thickness in diastole (LVAWd), and left ventricular posterior wall end diastole (LVPWd) were evaluated.

### Histological Analysis

The heart tissues were collected 7 days after operation, fixed in 4% paraformaldehyde (PFA), and made into paraffin-embedded slices. The hematoxylin and eosin (H&E) staining, Masson’s trichrome staining (Thermo, United States), and Prussian blue staining were determined according to manufacturer’s protocol, followed by observation under microscope (Leica, Germany).

### Oxygen-Glucose DeprivationModel

To establish *in vitro* MI model, H9c2 cells were placed in DMEM deprived of glucose (Thermo) and placed in a 37°C anaerobic atmosphere (1% O_2_ and 5% CO_2_) for 24 h (Du et al., 2020; Niu et al., 2020; Wu et al., 2021).

### Cell Viability

The viability of H9c2 cells was determined by cell counting kit 8 (CCK-8) following the manufacturer’s description. In brief, cells were placed in 96-well plates at a density of 5,000 cells per well, and treated with Res for 48 h, followed by incubation with CCK-8 reagent for another 1.5 h. The absorbance at 450 nm was checked by a microplate detector (Thermo).

### ELISA

The levels of inflammatory factors including IL-6 and IL-1β in heart tissues, serum, and culture medium of H9c2 cells were measured by ELISA assay using commercial IL-6 Rat ELISA Kit (BMS625, Invitrogen, United States) and IL-1β Rat ELISA Kit (BMS630, Invitrogen, United States) in accordance with manufacturer’s protocols.

### Western Blotting

The total proteins were extracted from heart tissues, or cells by using ice-cold RIPA lysis buffer (Beyotime, China). A total of 30 μg proteins were separated by SDS-polyacrylamide gel (SDS-PAGE), shifted to polyvinylidene difluoride (PVDF) membranes (Millipore, Germany), and blocked in 5% non-fat milk, followed by incubation with specific primary anti-collagen 1 (ab254113, Abcam, United States, 1:1,000), anti-α-SMA (ab40854, Abcam, United States, 1:1,000), anti-GPX4 (ab252833, Abcam, United States, 1:1,000), anti-SLC7A11 (ab175186, Abcam, United States, 1:1,000) antibodies at 4°C overnight. Next day, the protein bands were incubated with HRP conjugated secondary anti-mouse or anti-rabbit antibodies (ab175775/ab288151, Abcam, United States, 1:1,000) at room temperature for 1 h. The bands were then visualized by using ECL (Thermo) on a gel image system (Bio-Rad, United States). All antibodies were purchased from Abcam.

### Determination of Ferroptosis Biomarkers

The occurrence of ferroptosis was evaluated by levels of malondialdehyde (MDA), glutathione (GSH), lipid ROS, and iron. To determine lipid ROS, cells were labeled with C11-BODIPY (581/591) (5 µM) in dark for 30 min at 37°C, then were harvested for flow cytometry on FACs Calibur (BD Biosciences). The level of intracellular iron was measured by an Iron Assay Kit as per the manufacturer’s protocols. The MDA level was assessed by using an MDA assay kit (Sigma). To check GSH activity, cells were collected and lysed in a 5% 5-sulfosalicylic acid (SSA) solution and measured by a Reduced Glutathione Assay kit (BioVision) in line with the manufacturer’s instructions.

### Quantitative RT-PCR (qRT-PCR)

The heart tissues and H9c2 cells were homogenized with Trizol reagent (Thermo) to isolate total RNA. The RNA was reverse-transcribed to cDNA by using the PrimeScript First Strand cDNA Synthesis Kit (Takara, China), followed by quantification with SYBR Green Master Mix (Takara) using the 2-ΔΔCt method. The GAPDH was adopted as internal control for normalization of KAT5 and GPX4.

The primer sequences are as follows:

**Table:** 

**Gene**	**Primer**
KAT5	5′- GCG​GAG​GTG​GGG​GAG​AT-3′
	5′- AGGGTACGGGGAGAAGTACC-3′
GPX4	5′- GCA​ACC​AGT​TCG​GGA​GGC​AGG​AG-3′
	5′- CCTCCATGGGACCATAGCGCTTC-3′
GAPDH	5′-AAGAAGGTGGTGAAGCAGGC-3′
	5′-TCCACCACCCAGTTGCTGTA-3′

### Statistical Analysis

SPSS 20.0 software was adopted for statistical analysis. Data were shown as means ± standard deviation. Student’s *t* test or one-way analysis of variance with Dunnett’s multiple comparisons test were applied for comparison between two or multiple groups. The experiments were performed at least in triplicate.

## Results

### Res Relieves Myocardial Injury in MI Rats

**FIGURE 1:**
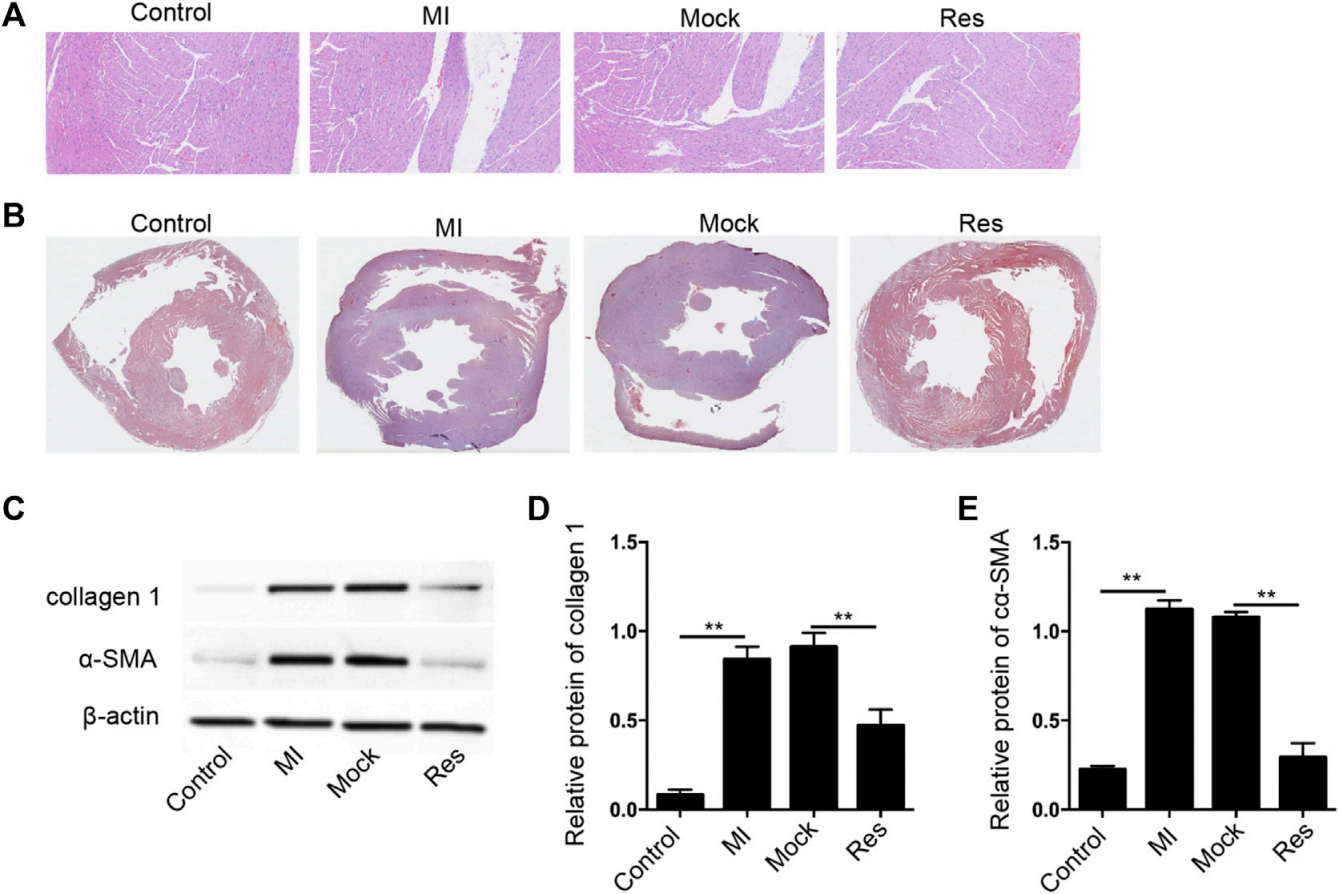
Res attenuates myocardial injury in MI rats **(A–E)** The MI rats were injected with Res (*n* = 5 for each group). **(A)** The myocardium injury was analyzed by Hematoxylin & Eosin (H&E) staining **(B)** The myocardial fibrosis was measured by Masson’s staining. **(C–E)** The expression of collagen 1 and α-SMA was detected by Western blot analysis and was normalized on the expression of β-actin. Data are presented as mean ± SD. Statistic significant differences were indicated: **p < 0.01.

In order to assess the impact of Res on MI-induced myocardial dysfunction, the MI rat model was constructed and was injected with Res. The treatment of Res did not affect the body weight of the rats, suggesting that there was no toxicological effect on the rats (data bot shown). We observed that the treatment of Res attenuated the MI-related myocardium injuries in the rats (Figure 1A). The myocardial fibrosis was induced in the MI rats and the treatment of Res relieved the phenotype (Figure 1B). Meanwhile, the expression of collagen one and α-SMA was induced in MI rats, in which the treatment of Res could decrease the expression (Figures 1C–E).

### Res Alleviates Inflammation and Ferroptosis in MI Rats

**FIGURE 2:**
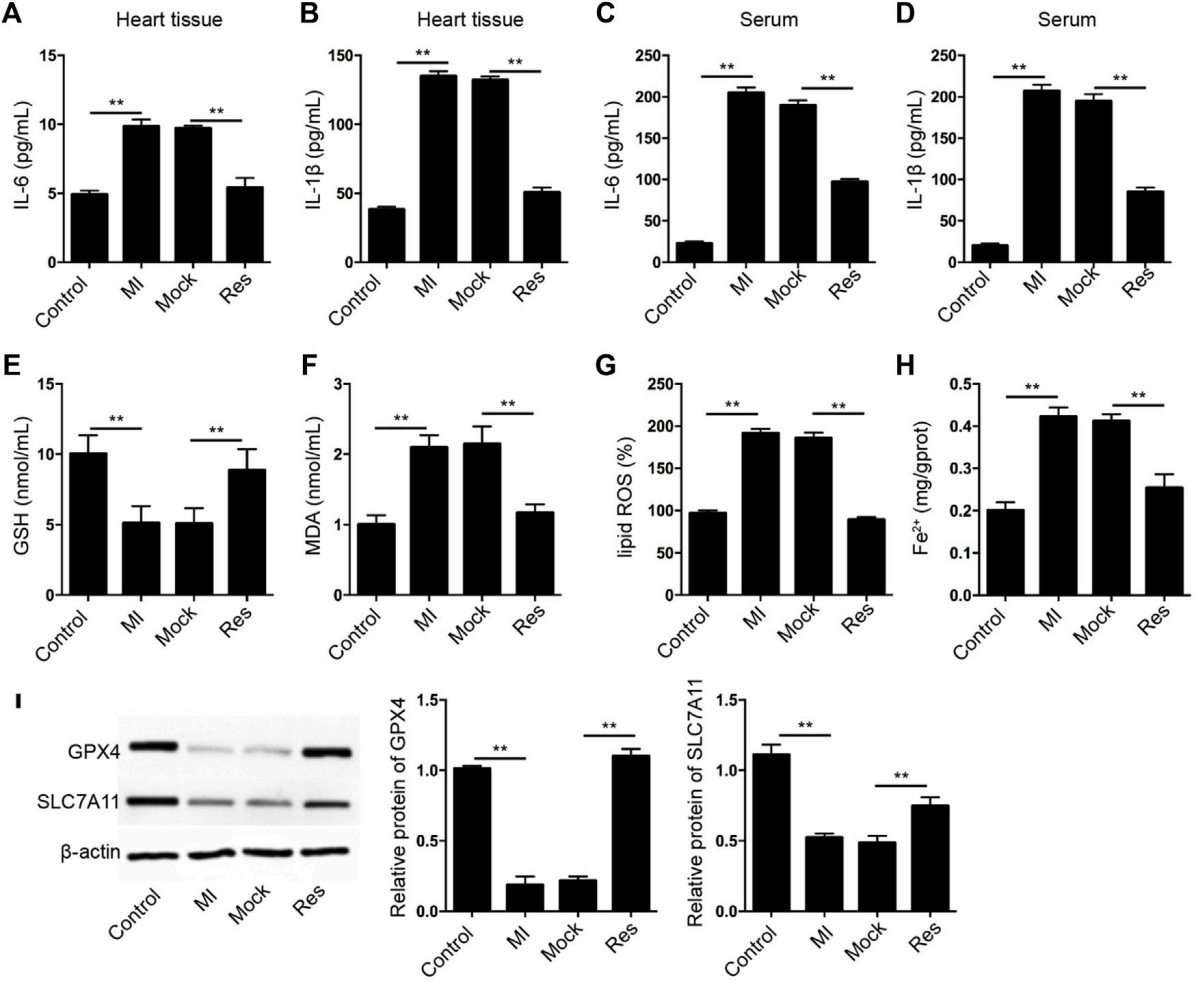
**Res alleviates inflammation and ferroptosis in MI rats. (A–I)** The MI rats were injected with Res (*n* = 5 for each group). **(A–D)** The levels of IL-6 and IL-1β were measured by ELISA assay in heart tissues and serum samples of the rats. The levels of GSH **(E)**, MDA **(F)**, lipid ROS **(G)**, and Fe^2+^ **(H)** were analyzed. **(I)** The expression of GPX4 and SLC7A11 was detected by Western blot analysis and was normalized on the expression of β-actin. Data are presented as mean ± SD. Statistic significant differences were indicated: **p < 0.01.

Next, we further assessed the effect of Res on inflammation and ferroptosis in MI rats. We identified that treatment of Res suppressed the levels of IL-6 and IL-1β in heart tissues and serum samples of the MI rats (Figures 2A–D). Importantly, the GSH levels were inhibited and MDA, lipid ROS, and Fe^2+^ levels were induced in MI rats in which the treatment of Res could reverse the phenotypes (Figures 2E–H). Meanwhile, the expression of GPX4 and SLC7A11 was reduced in MI rats, while the treatment of Res could rescue the expression in the model (Figure 2I).

### Res Relieves OGD-Induced Cardiomyocyte Injury

**FIGURE 3:**
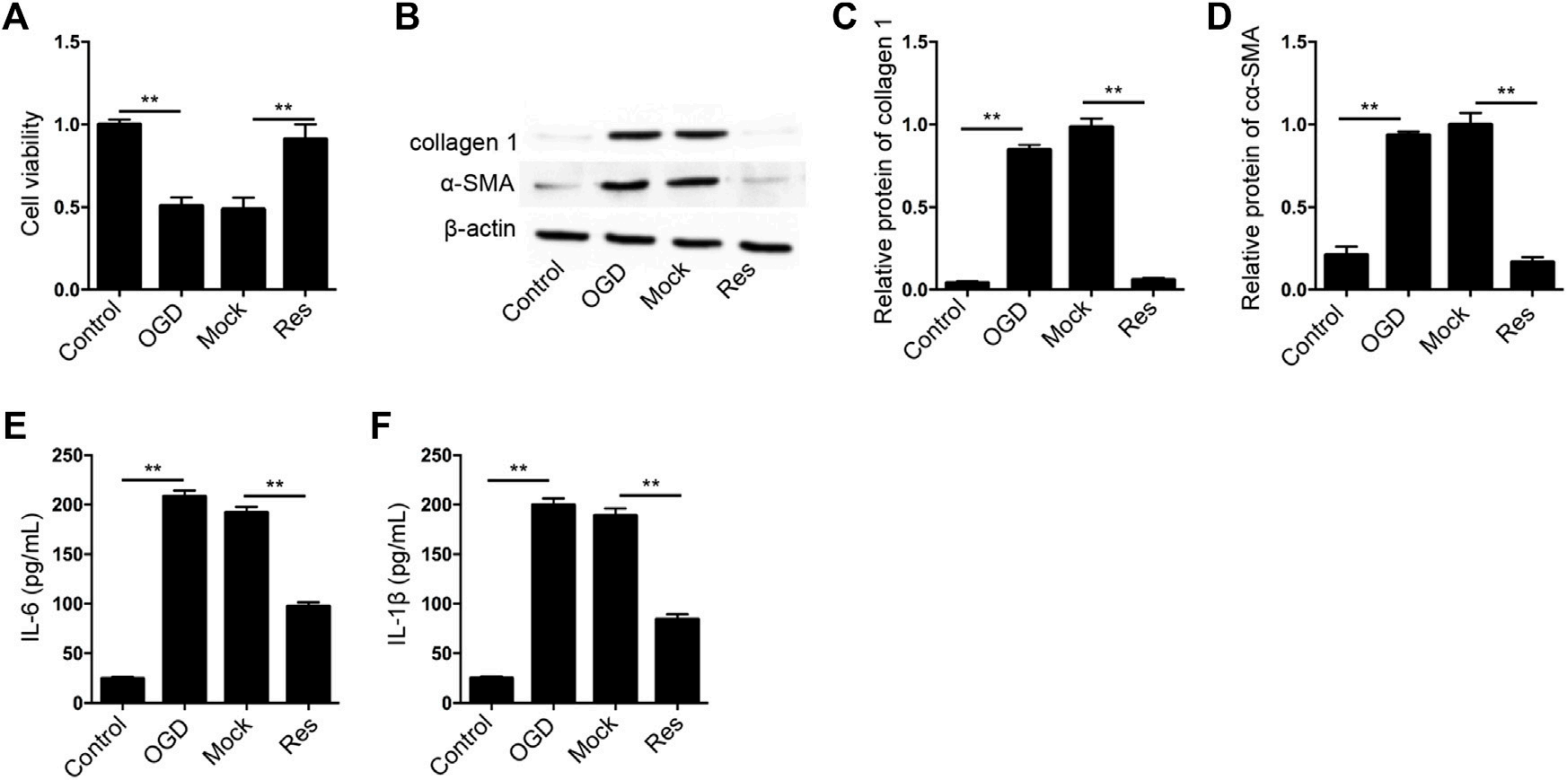
Res relieves OGD-induced cardiomyocyte injury. **(A–F)** The OGD-treated H9c2 cells were treated with Res. **(A)** The cell viability was measured by CCK-8 assay. **(B–D)** The expression of collagen 1 and α-SMA was tested by Western blot analysis and was normalized on the expression of β-actin. **(E,F)** The levels of IL-6 and IL-1β were detected by ELISA. Data are presented as mean ± SD. Statistic significant differences were indicated: **p < 0.01.

Next, we further evaluated the effect of Res on OGD-induced cardiomyocyte injury *in vitro*. The cell viability was reduced in OGD-treated H9c2 cells but the treatment of Res rescued the phenotype in the cells (Figure 3A). The expression of collagen 1 and α-SMA was enhanced by OGD treatment, while the treatment of Res suppressed the expression in H9c2 cells (Figures 3B–D). In addition, the OGD-induced IL-6 and IL-1β levels were attenuated by the treatment of Res in H9c2 cells (Figures 3E,F).

### Res Represses OGD-Induced Cardiomyocyte Ferroptosis

**FIGURE 4:**
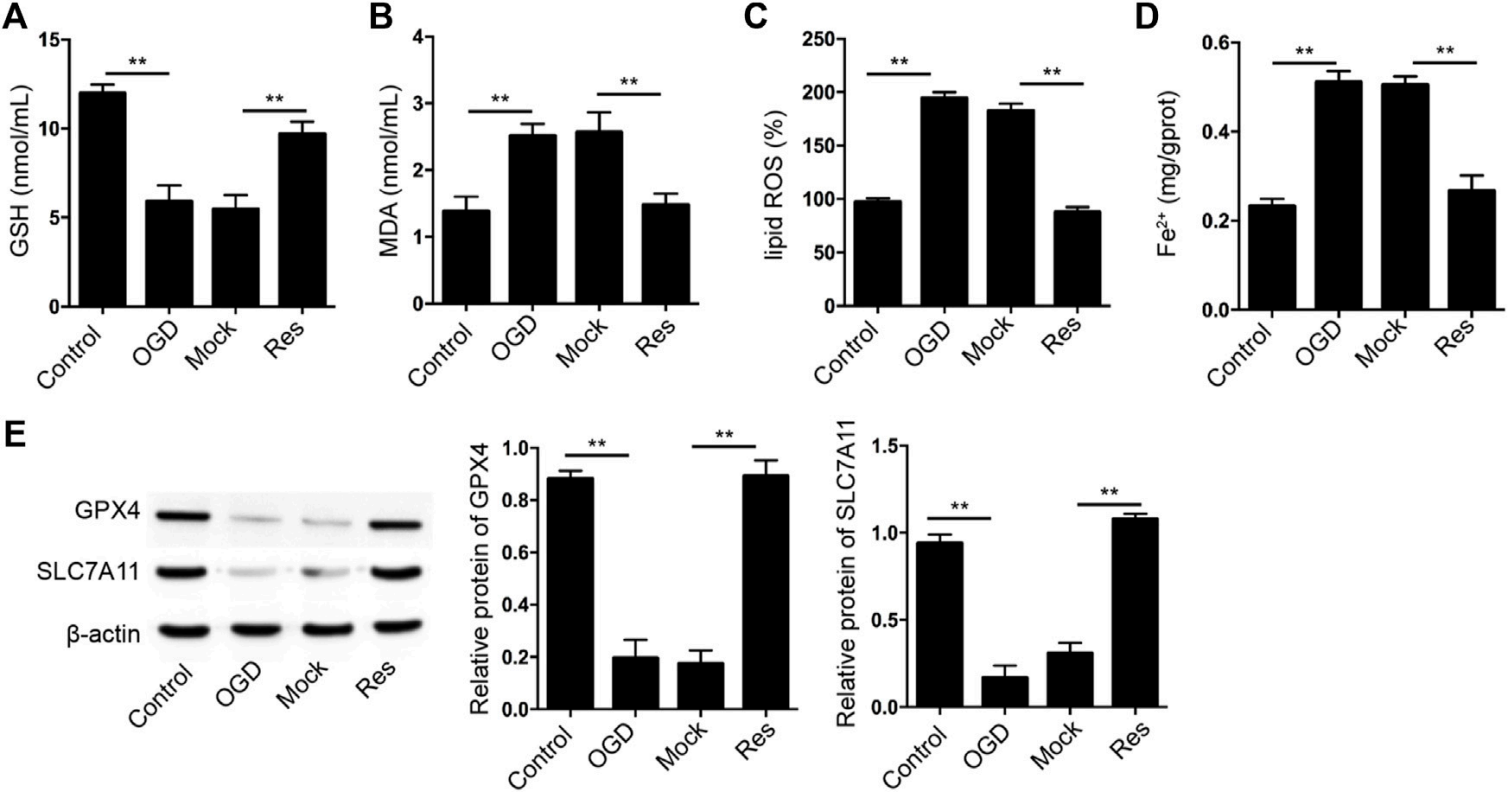
Res represses OGD-induced cardiomyocyte ferroptosis**. (A–E)** The OGD-treated H9c2 cells were treated with Res. The levels of GSH **(A)**, MDA **(B)**, lipid ROS **(C)**, and Fe^2+^ **(D)** were analyzed. **(E)** The expression of GPX4 and SLC7A11 was detected by Western blot analysis and was normalized on the expression of β-actin. Data are presented as mean ± SD. Statistic significant differences were indicated: **p < 0.01.

Then, we further evaluated the effect of Res on the ferroptosis of cardiomyocytes. The GSH levels were decreased and MDA, lipid ROS, and Fe2+ levels were increased in OGD-treated H9c2 cells, in which the treatment of Res was able to reverse the phenotypes (Figures 4A–D). Besides, the expression of GPX4 and SLC7A11 was repressed in OGD-treated H9c2 cells, while the treatment of Res enhanced the expression in the cells (Figure 4E).

### Res Enhances GPX4 Expression by Inducing KAT5 in Cardiomyocytes

**FIGURE 5:**
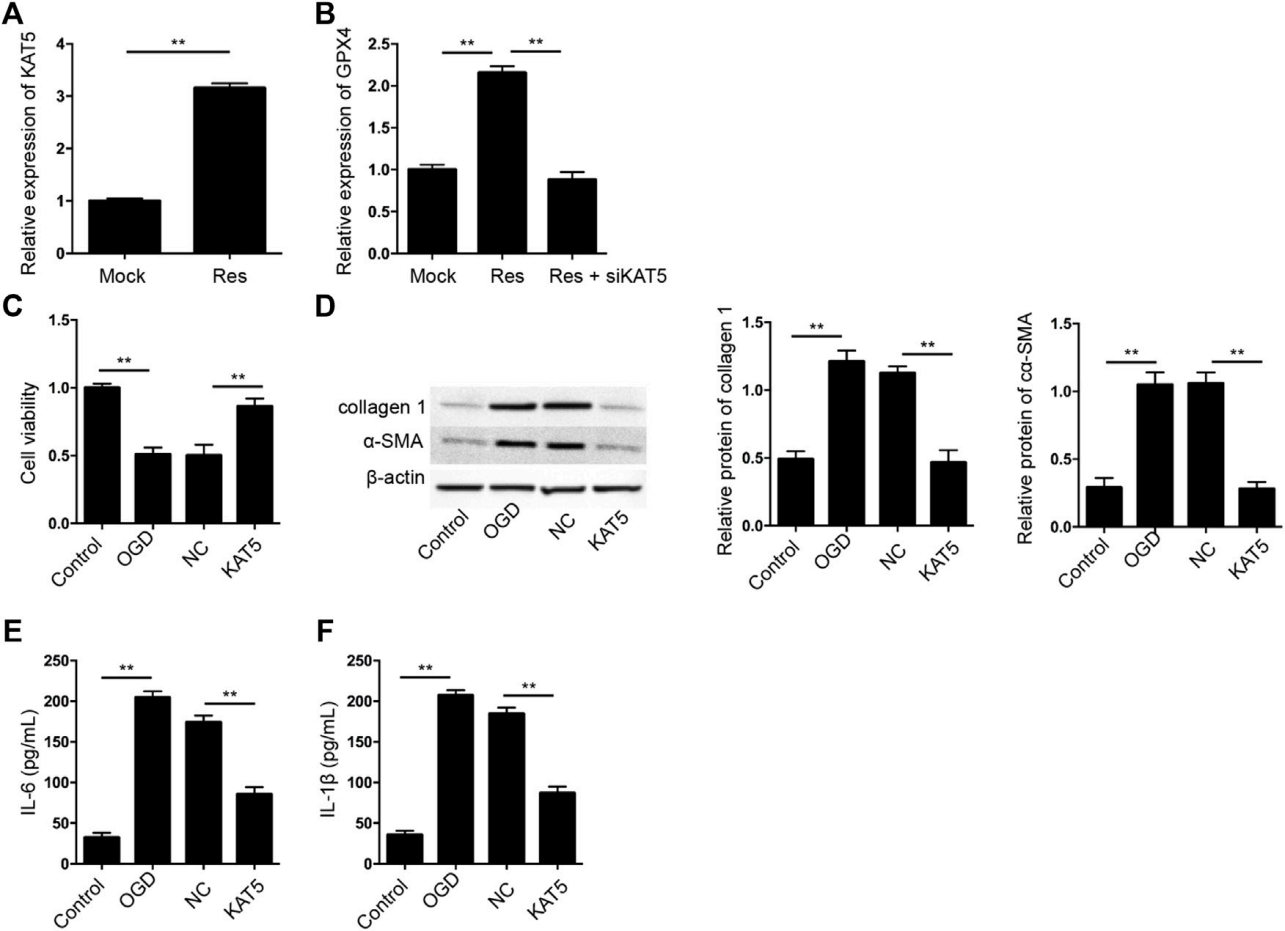
Res enhances GPX4 expression by inducing KAT5 in cardiomyocytes. **(A)** The expression of KAT5 was measured by qPCR in H9c2 cells treated with Res. **(B)** The expression of GPX4 was determined by qPCR in H9c2 cells treated with Res and KAT5 siRNA. **(C–F)** The OGD-treated H9c2 cells were treated with KAT5 overexpressing plasmid. **(C)** The cell viability was measured by CCK-8 assay. **(D)** The expression of collagen 1 and α-SMA was tested by Western blot analysis and was normalized on the expression of β-actin. **(E,F)** The levels of IL-6 and IL-1β were detected by ELISA. Control, H9c2 without OGD treatment; NC, empty plasmid. Data are presented as mean ± SD. Statistic significant differences were indicated: **p < 0.01.

Next, we tried to explore the mechanism of Res protection against cardiomyocyte injury. We identified that the treatment of Res was able to induce KAT5 expression in H9c2 cells (Figure 5A). Meanwhile, the treatment of Res enhanced GPX4 expression, but the depletion of KAT5 could reverse the effect of Res in H9c2 cells (Figure 5B). We then verified the effect of KAT5 on OGD-induced cardiomyocyte injury *in vitro*. The cell viability was reduced in OGD-treated H9c2 cells but the overexpression of KAT5 rescued the phenotype in the cells (Figure 5C). The expression of collagen 1 and α-SMA was enhanced by OGD treatment, while the overexpression of KAT5 suppressed the expression in H9c2 cells (Figure 5D). Besides, the OGD-induced IL-6 and IL-1β levels were attenuated by the overexpression of KAT5 in H9c2 cells (Figures 5E,F).

### KAT5 Reduces OGD-Induced Cardiomyocyte Ferroptosis

**FIGURE 6:**
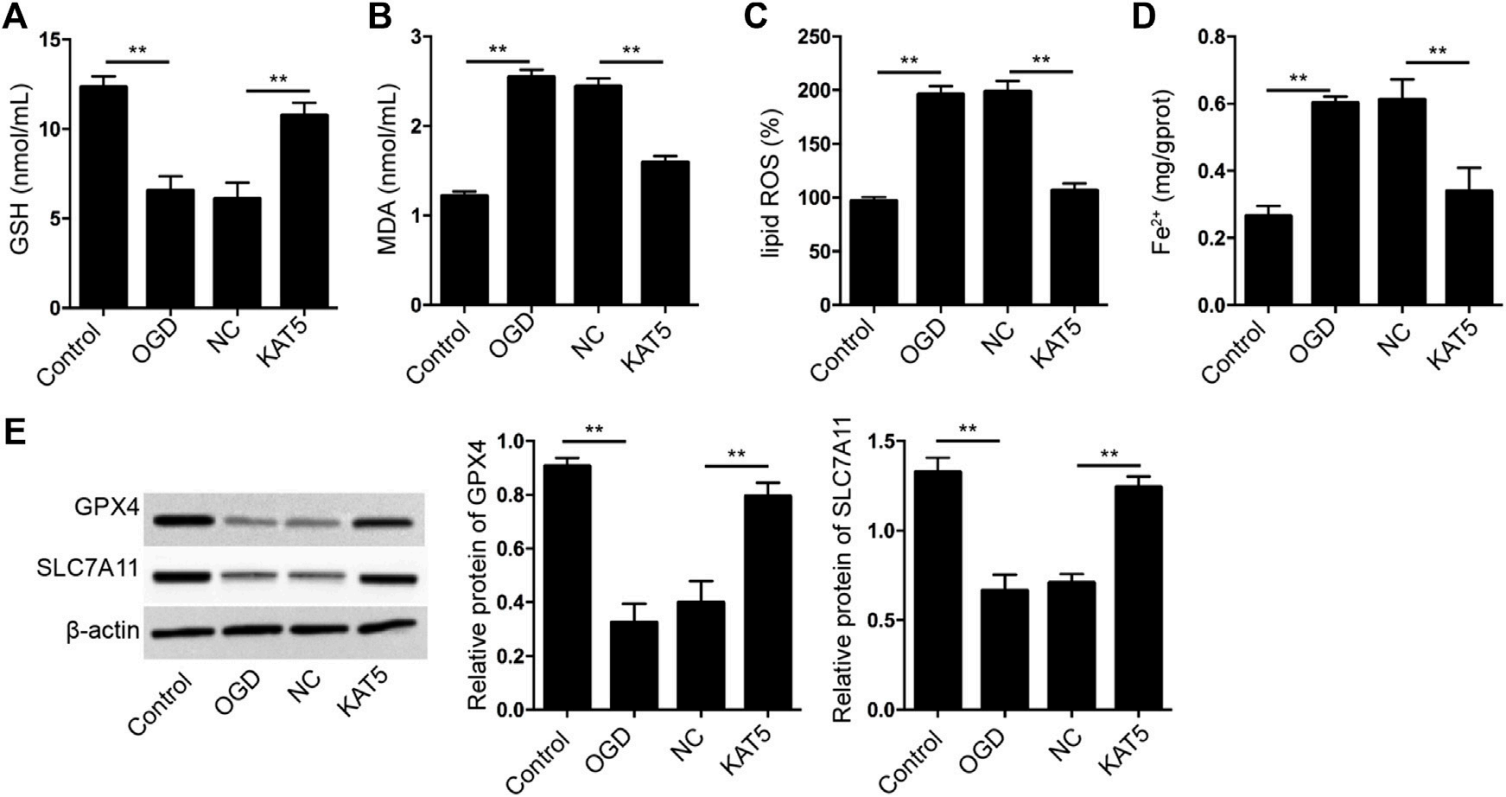
KAT5 reduces OGD-induced cardiomyocyte ferroptosis**. (A–E)** The OGD-treated H9c2 cells were treated with KAT5 overexpressing plasmid. The levels of GSH **(A)**, MDA **(B)**, lipid ROS **(C)**, and Fe^2+^ **(D)** were analyzed. **(E)** The expression of GPX4 and SLC7A11 was detected by Western blot analysis and was normalized on the expression of β-actin. Data are presented as mean ± SD. Statistic significant differences were indicated: **p < 0.01.

Next, we further assessed the effect of KAT5 on the ferroptosis of cardiomyocytes. The GSH levels were reduced and MDA, lipid ROS, and Fe2+ levels were enhanced in OGD-treated H9c2 cells, in which the overexpression of KAT5 was able to reverse the phenotypes (Figures 6A–D). In addition, the expression of GPX4 and SLC7A11 was suppressed in OGD-treated H9c2 cells, while the overexpression of KAT5 rescued the expression in the cells (Figure 6E).

### Res Relieves OGD-Induced Cardiomyocyte Injury by KAT5/GPX4 Signaling

**FIGURE 7:**
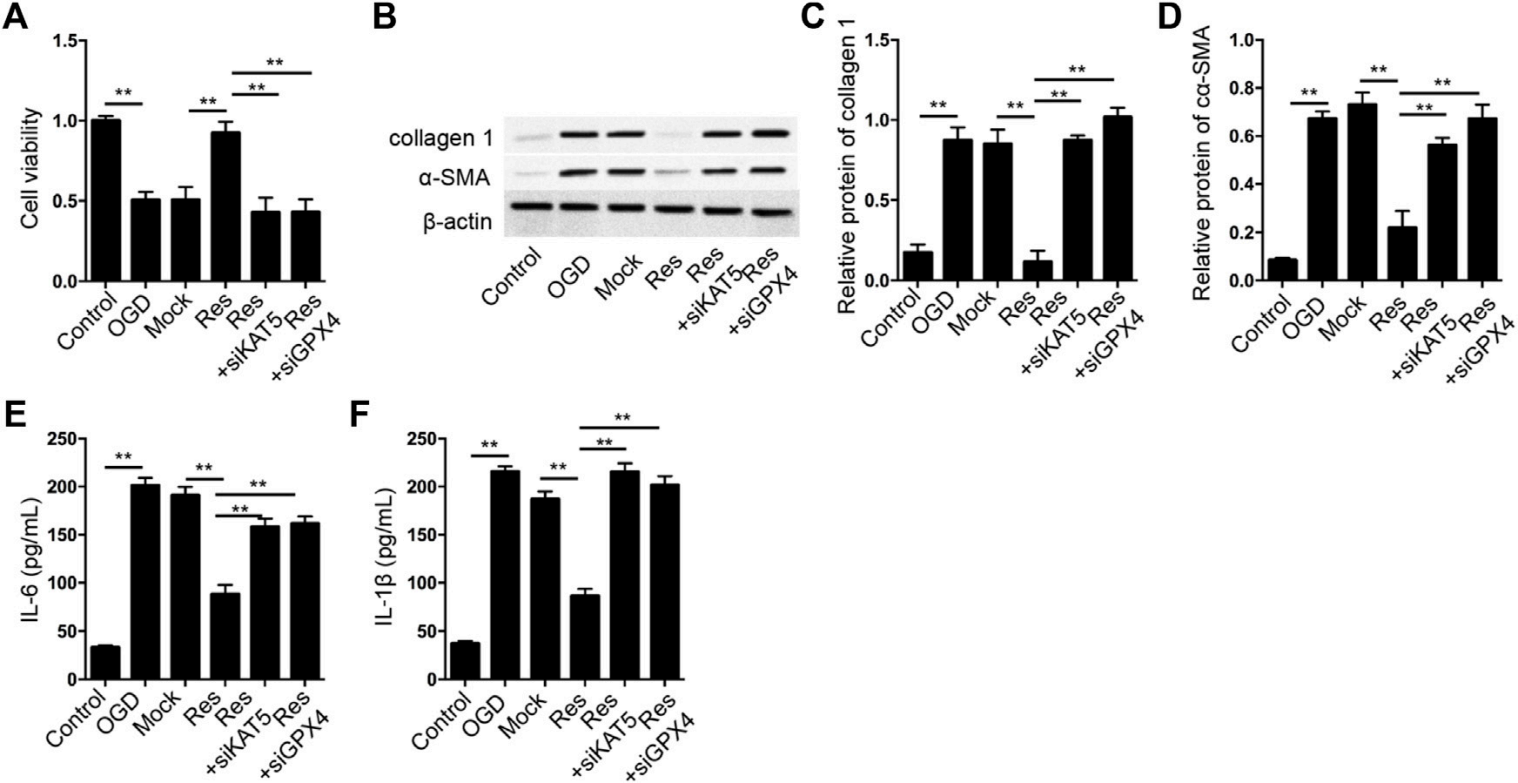
Res relieves OGD-induced cardiomyocyte injury by KAT5/GPX4 signaling**. (A–F)** The OGD-treated H9c2 cells were treated with Res, or co-treated with GPX4 or SLC7A11 siRNA. **(A)** The cell viability was measured by CCK-8 assay. **(B–D)** The expression of collagen 1 and α-SMA was tested by Western blot analysis and was normalized on the expression of β-actin. **(E,F)** The levels of IL-6 and IL-1β were detected by ELISA. Data are presented as mean ± SD. Statistic significant differences were indicated: **p < 0.01.

Next, we analyzed the correlation of Res/KAT5/GPX4 axis with OGD-induced cardiomyocyte injury *in vitro*. The cell viability was induced by the treatment of Res in OGD-treated H9c2 cells, in which the depletion of KAT5 or GPX4 could attenuate the phenotype (Figure 7A). The expression of collagen 1 and α-SMA was reduced by Res in OGD-treated H9c2 cells, but the knockdown of KAT5 or GPX4 rescued the expression (Figures 7B–D). In addition, the levels of IL-6 and IL-1β were inhibited by Res, while the silencing of KAT5 or GPX4 could reverse the effect in OGD-treated H9c2 cells (Figures 7E,F).

Moreover, we validated that the ferroptosis inhibitor Fer-1 was able to inhibit OGD-induced H9c2 cell injury, inflammation, and ferroptosis, while the depletion of KAT5 or GPX4 could reverse the effect of Fer-1 in the model (Figure S1).

## Discussion

MI serves as a coronary artery-related disease and is a leading cause of sudden death globally. Res is a bioactive component and has presented antioxidant, anti-inflammatory and anti-microbial properties. Nevertheless, the impact of Res on ferroptosis in MI progression is still unclear. In the present study, we discovered the effect of Res on ferroptosis and myocardial injury in MI.

The function of Res in myocardial injury has been reported in several studies. It has been reported that Res attenuates ischemia-reperfusion injury by enhancing autophagy in diabetic myocardium (Qu et al., 2019). Res relieves isoproterenol-induced MI by AMPK/eNOS/NO signaling in rats (Feng et al., 2019). Res attenuates CIH-induced myocardial injury through regulating Nrf2 expression and the activation of NLRP3 inflammasome (Sun et al., 2020). Res induces myocardial Nrf2 expression to attenuate myocardial ischemia/reperfusion injury in type 2 diabetic rats (Xu et al., 2019b). Res suppresses ischemia-induced myocardial senescence by targeting the activation of NLRP3 inflammasome (Feng et al., 2020). Ferroptosis plays crucial role in MI progression and has been identified as a therapeutic target in MI. Icariin represses hypoxia-reoxygenation-regulated ferroptosis of cardiomyocytes by targeting Nrf2/HO-1 signaling (Liu et al., 2021). Human umbilical cord blood-derived MSCs exosomes relieves myocardial injury *via* repressing ferroptosis in MI mice (Song et al., 2021). MiR-30d suppresses cardiomyocyte autophagy and inducing ferroptosis in MI (Tang et al., 2020). Oxidative stress plays crucial roles in the process of ferroptosis (Lillo- Moya et al., 2021). The antioxidant effects of Res contribute substantially to the health benefits and the biomedical activities of Res (Xia et al., 2017). In this study, we found that the treatment of Res attenuated the MI-related myocardium injury, inflammation, fibrosis in the rats. Meanwhile, the GSH levels were inhibited and MDA, lipid ROS, and Fe^2+^ levels were induced in MI rats, in which the treatment of Res could reverse the phenotypes. The expression of GPX4 and SLC7A11 was reduced in MI rats, while the treatment of Res could rescue the expression in the model. These data present a novel function of Res in MI-related cardiac dysfunction, providing valuable evidence for the potential therapeutic role of Res in MI. In addition, we administrated Res directly around the infarction, like some previous studies (Li et al., 2021a), and some studies apply the intraperitoneal injection (He et al., 2021), the effectiveness needs to be compared between these two manner in future investigations.

Meanwhile, it has been reported that KAT5 enhances cell-cycle arrest and DNA damage response in neonatal cardiomyocytes (Wang et al., 2021). The Egr-1/miR-15a-5p/GPX4 signaling modulates ferroptosis in acute MI (Fan et al., 2021). The downregulation of GPX4 induces ferroptosis of cardiomyocytes during MI progression (Park et al., 2019). Interestingly, a recent study reported that Res attenuates myocardial ischemia-reperfusion injury by targeting ferroptosis *via* by the mechanism of ubiquity specific peptidase 19 (USP19)-Beclin1-mediated autophagy (Li et al., 2022). Moreover, it has been reported that Ketamine inhibits proliferation and promotes ferroptosis of by targeting KAT5/GPX4 signaling in breast cancer cells (Li et al., 2021b). In our study, we identified that Res alleviated MI-induced myocardial injury by modulating ferroptosis *via* the mechanism of KAT5-induced epigenetic regulation of GPX4. We confirmed that KAT5 alleviated OGD-induced cardiomyocyte injury and ferroptosis. The depletion of KAT5 or GPX4 could reverse the effect of Res on OGD-induced cardiomyocyte injury. Our data suggest that Res attenuates cardiomyocyte injury by targeting KAT5/GPX4 signaling.

In summary, we concluded that Res attenuated myocardial injury by inhibiting ferroptosis *via* inducing KAT5/GPX4 in myocardial infarction. Our finding provides new evidence of the potential therapeutic effect of Res on MI by targeting ferroptosis.

## Data Availability

The raw data supporting the conclusion of this article will be made available by the authors, without undue reservation.
